# Anti-proliferative effect of methanolic extract of *Gracilaria tenuistipitata* on oral cancer cells involves apoptosis, DNA damage, and oxidative stress

**DOI:** 10.1186/1472-6882-12-142

**Published:** 2012-08-31

**Authors:** Chi-Chen Yeh, Jing-Iong Yang, Jin-Ching Lee, Chao-Neng Tseng, Ya-Ching Chan, You-Cheng Hseu, Jen-Yang Tang, Li-Yeh Chuang, Hurng-Wern Huang, Fang-Rong Chang, Hsueh-Wei Chang

**Affiliations:** 1 Graduate Institute of Natural Products, College of Pharmacy, Kaohsiung Medical University, Kaohsiung, Taiwan; 2 Department of Seafood Science, National Kaohsiung Marine University, Kaohsiung, Taiwan; 3 Department of Biotechnology, Kaohsiung Medical University, Kaohsiung, Taiwan; 4 Department of Biomedical Science and Environmental Biology, Kaohsiung Medical University, Kaohsiung, Taiwan; 5 Department of Cosmeceutics, College of Pharmacy, China Medical University, Taichung, Taiwan; 6 Department of Radiation Oncology, Faculty of Medicine, College of Medicine, Kaohsiung Medical University, Kaohsiung, Taiwan; 7 Department of Radiation Oncology, Kaohsiung Medical University Hospital, Kaohsiung, Taiwan; 8 Department of Chemical Engineering, I-Shou University, Kaohsiung, Taiwan; 9 Institute of Biomedical Science, National Sun Yat-Sen University, Kaohsiung, Taiwan; 10 Center of Excellence for Environmental Medicine, Cancer Center, Kaohsiung Medical University Hospital, Kaohsiung Medical University, Kaohsiung, Taiwan

**Keywords:** Red algae, Oral cancer, Apoptosis, γ-H2AX, ROS, Mitochondrial membrane potential, Glutathione

## Abstract

**Background:**

Methanolic extracts of *Gracilaria tenuistipitata* (MEGT) were obtained from the edible red algae. Previously, we found that water extract of *G. tenuistipitata* was able to modulate oxidative stress-induced DNA damage and its related cellular responses.

**Methods:**

In this study, the methanol extraction product MEGT was used to evaluate the cell growth inhibition in oral cancer cells and its possible mechanism was investigated.

**Results:**

The cell viability of MEGT treated Ca9-22 oral cancer cell line was significantly decreased in a dose–response manner (*p* < 0.05). The sub-G1 population and annexin V intensity of MEGT-treated Ca9-22 cancer cells were significantly increased in a dose–response manner (*p* < 0.0005 and *p* < 0.001, respectively). The γH2AX intensities of MEGT-treated Ca9-22 cancer cells were significantly increased in a dose–response manner (*p* < 0.05). The reactive oxygen species (ROS) and glutathione (GSH)-positive intensities of MEGT-treated Ca9-22 oral cancer cells were significantly increased and decreased, respectively, in a dose–response manner (*p* < 0.05). The DiOC_2_(3) intensity for mitochondrial membrane potential (MMP) of MEGT-treated Ca9-22 cancer cells was significantly decreased in a dose–response manner (*p* < 0.05).

**Conclusions:**

These results indicated that MEGT had apoptosis-based cytotoxicity against oral cancer cells through the DNA damage, ROS induction, and mitochondrial depolarization. Therefore, MEGT derived from the edible algae may have potential therapeutic effects against oral squamous cell carcinoma (OSCC).

## Background

Oral squamous cell carcinoma (OSCC), the sixth most common cancer worldwide [1], is characterized by high morbidity and mortality rates. The poor prognosis for OSCC [2] is mainly due to its poor response to chemotherapy [3]. Therefore, drug discovery for anti-OSCC therapy remains challenging.

Natural products [4-8], especially from marine sources [9-12], offer an abundance for drug development to treat many types of cancer. Algae contain many bioactive primary and secondary metabolites [13,14] and represent about 9% of marine biomedical compounds [15]. Among them, the bioactive compounds of the genus *Gracilaria* had been summarized [16,17] and mainly classified by water [18-20] and ethanol/methanol [21-23] extractions. Most of these studies focus on health promoting effects, such as anti-inflammatory, anti-hypercholesterolemic, antioxidative, and antimicrobial properties rather than on cancer therapy.

The *Gracilaria* algae is inexpensive in Taiwan because it has been cultivated since 1961 [24], and therefore, is an abundant resource for research purposes. In our previous work [25], water extracts of *G. tenuistipitata* demonstrated a potential protective effect from hydrogen peroxide-induced DNA damage. However, extraction methods may influence the biological effects for some natural products including soy products [26]. Recently, many ethanolic or methanolic extracts of natural products were found to possess antiproliferative effects in cancer; such as Njavara ethanolic extracts for glioma cells [27], *Cassia tora* methanolic leaf extracts for HeLa cells [28], *Olea europaea* ethanolic extracts for leukemic cells [29], *Plocamium telfairiae* methanolic extracts for colon cancer cells [30], and *Indigofera linnaei* Ali methanolic extracts for tumor cells [31]. Accordingly, the biological effects for methanolic extracts of *G. tenuistipitata* (MEGT) were evaluated in this study.

In this study, we propose that MEGT has the potential to modulate the cell proliferation of OSCC. To test this hypothesis, the anticancer potential against the human OSCC cancer cells (Ca9-22) was explored in terms of the cell viability and the alterations of cell cycle, apoptosis, ROS, GSH content, and mitochondrial membrane potential to determine the possible mechanism of action.

## Methods

### Raw materials

The seaweed *Gracilaria tenuistipitata* was collected in spring of 2009 from a culture farm at Kouhu beach, Yunlin County, Taiwan, and was delivered to the laboratory at 0°C. In the laboratory, the algae were washed with running tap water to remove epiphytes and encrusting material, soaked in distilled water twice, and then lyophilized. The dried sample was pulverized and passed through 60-mesh sieve. The lyophilized sample was then ground to a fine powder and stored at −40°C.

### Sample extraction

The dried samples (50 g) were immersed in 250 ml methanol three times and were immediately extracted with 1000 ml of 99.9% methanol in a mechanical shaker at room temperature for 24 h. Subsequently, the extract of methanol solution was filtered with Whatman No. 1 filter paper three times. The filtered solution was then collected and evaporated to dryness at 40 ± 2°C in a rotary evaporator (Buchi Laboratoriums-Technik, Switzerland). The dry extract was stored in a sealed container at −40°C until use.

### Cell cultures

The human OSCC cancer cell line, Ca9-22 [32,33], was cultured in DMEM medium (Gibco, Grand Island, NY, USA) and supplemented with 10% fetal bovine serum (FBS), 100 U/ml penicillin, 100 μg/ml streptomycin, 0.03% glutamine and 1 mM sodium pyruvate. The cells were incubated at 37°C in a humidified atmosphere containing 5% CO_2_.

### Cell viability assay

MEGT was dissolved in DMSO and added to medium to make the final concentration of DMSO less than 1%. Cell proliferation was determined by the WST-1 kit (Roche) [34]. Cells were plated at a density of 1 × 10^5^ cells/well in a 96-well cell culture plate and treated with methanolic extract at doses of 0.1, 0.25, 0.5, 1 mg/ml for 24 h. After incubation, the WST-1 proliferation reagent (Roche) was added to cells (10 μl per well) and continued to incubate for 1–2 h at 37°C. Plates were checked visually by comparing the colors of the wells with or without MEGT-treated cells.

### Cell cycle distribution

Cells were treated with a solvent vehicle of 0.1, 0.25, 0.5 and 1 mg/ml of MEGT for 24 h. After trypsinization, the cells were harvested, washed twice with PBS, and fixed overnight with 70% ethanol. After centrifugation, the cell pellets were stained with 10 μg/ml propidium iodide (PI) (Sigma, St Louis, MO, USA) and 10 μg/ml RNase A in PBS buffer for 15 min at room temperature in the dark. PI intensities were measured using a FACSCalibur flow cytometer (Becton-Dickinson, Mansfield, MA, USA) and analyzed Win-MDI software, version 2.9 (http://facs.scripps.edu/wm29w98.exe).

### Flow cytometry-based detection of Annexin V staining

Annexin V staining (Pharmingen, San Diego, CA) was performed to examine the apoptosis status as previously described [35]. A total of 1 × 10^6^ cells per 100-mm petri-dish were treated with vehicle or increasing concentrations of MEGT for 24 h. Finally, cells were stained with 10 μg/ml of annexin V-fluorescein isothiocyanate (FITC) and analyzed using the FACSCalibur flow cytometer.

### Flow cytometry-based detection of γ-H2AX staining

The MEGT-treated cells were fixed in 70% ethanol, washed twice in BSA-PBST solution (1% bovine serum albumin and 0.2% Triton X-100 in PBS; Sigma), and incubated with 100 μl of BSA-PBST solution containing 0.2 μg p-Histone H2A.X (Ser 139) monoclonal antibody (Santa Cruz Biotechnology, Santa Cruz, CA, USA) overnight at 4°C. After washing twice with BSA-PBST, cells were suspended in Alexa Fluor 488-tagged secondary antibody (Jackson Laboratory, Bar Harbor, ME, USA) at a 1:100 dilution for 1 h. After washing twice with BSA-PBST, cells were resuspended in PBS for analysis using the FACSCalibur flow cytometer.

### Flow cytometry-based detection of intracellular reactive oxygen species (ROS)

Intracellular ROS was measured using 2',7'-dichlorodihydrofluorescein diacetate (DCFH-DA) as previously described [36] with slight modification. MEGT-treated cells were washed with PBS twice and then incubated with 10 μM H2DCF-DA in PBS for 30 min at 37°C. Cells were harvested and washed twice with PBS. After centrifugation, cells were resuspended in PBS and immediately forwarded to analysis by the FACSCalibur flow cytometer with excitation and emission settings of 480 and 525 nm, respectively.

### Flow cytometry-based detection of cellular reduced glutathione (GSH) content

Cells (8 × 10^5^) were cultured in 60-mm tissue-culture dishes for 24 h. The culture medium was replaced with new medium when the cells were 80% confluent. After MEGT treatments, the cells were incubated with 5 μM CMF-DA for 20 min at 37°C in the CO_2_ incubator. The CMF fluorescence is directly related to the intracellular GSH level. After CMF-DA staining, the cells were washed with PBS, collected by centrifugation, and then measured with the FACS-Calibur flow cytometer.

### Flow cytometry-based detection of mitochondrial membrane potential

Mitochondrial membrane potential (MMP) was measured using a MitoProbe™^TM^ DiOC_2_(3) assay kit (Invitrogen, San Diego, CA, USA). MEGT-treated Ca9-22 cells were suspended in 1 ml of warm PBS at approximately 1 × 10^6^ cells/ml, loaded with 5 μl of 10 μM DiOC_2_(3), and incubated at 37°C, 5% CO_2_, for 20 to 30 min. Cells were then harvested, washed and resuspended in PBS and analyzed immediately using flow cytometry assay with excitation and emission settings of 488 and 525 nm, respectively.

### Statistical analysis

All data are presented as means ± S.E. Comparison between experimental groups was assessed by one-way ANOVA with Tukey's HSD Post Hoc Test using the software JMP® 9. Differences between treatments of different concentrations containing the same letter are not significant.

## Results

### Cytotoxicity effects of MEGT-treated Ca9-22 oral cancer cells

**Figure 1:**
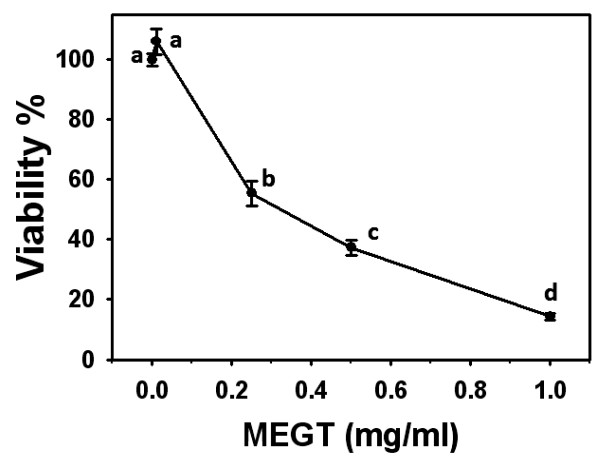
**Proliferation of Ca9-22 oral cancer cells is inhibited by methanolic extracts of *G. tenuistipitata *(MEGT).** Cells were incubated with various concentrations of MEGT (0, 0.01, 0.25, 0.5, and 1 mg/ml) for 24 h. Cell viability was determined by WST-1 assay. Data are expressed as mean ± S.E. (n = 3). Differences between treatments of different concentrations containing the same letter are not significant.

In the WST-1 assay (Figure 1), the relative cell viability at various concentrations of MEGT (0, 0.01, 0.25, 0.5 and 1 mg/ml) after 24 h were 100.0 ± 2.1, 106.1 ± 4.3, 55.5 ± 4.1, 37.4 ± 2.5, 14.2 ± 1.1 (n = 5). The cell viability of MEGT-treated Ca9-22 oral cells significantly decreased in a dose–response manner (*p* < 0.05). The IC_50_ value of the MEGT-treated Ca9-22 oral cancer cells at 24 h was 0.326 mg/ml.

### Changes of cell cycle distribution in MEGT-treated Ca9-22 oral cancer cells

**Figure 2:**
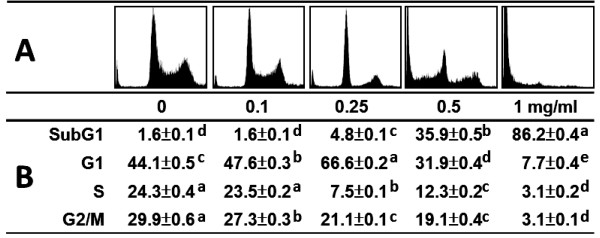
**Methanolic extracts of *G. tenuistipitata *(MEGT) induces an accumulation in the sub-G1 population in Ca9-22 oral cancer cells.** Cells were treated with the indicated 0, 0.01, 0.25, 0.5, and 1 mg/ml of MEGT for 24 h, respectively. (**A**) Cell cycle profiles for MEGT-treated Ca9-22 oral cancer cells and vehicle controls at 24 h. (**B**) The quantification analysis for the percentages of cell cycle stages. Data are presented as mean ± S.E. (n = 3). Differences between treatments of different concentrations containing the same letter are not significant.

The G1 percentages were significantly increased at the concentrations from 0 to 0.25 mg/ml MEGT leading to G1 arrest (Figure 2). Concentrations greater than 0.5 mg/ml significantly decreased the G1 percentages of cells (*p* < 0.0005). In addition, the sub-G1 populations of MEGT-treated cells were slightly increased at a dose of 0.25 mg/ml, moderately increased at 0.5 mg/ml, and dramatically increased at 1 mg/ml. This MEGT-induced sub-G1 accumulation of Ca9-22 oral cancer cells was significantly increased in a dose–response manner (*p* < 0.0005).

### Apoptosis induction of MEGT-treated Ca9-22 oral cells

**Figure 3:**
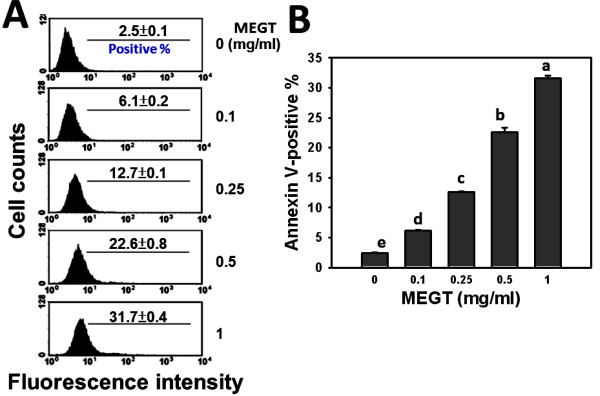
**Methanolic extracts of *G. tenuistipitata *(MEGT) induced apoptosis of Ca9-22 oral cancer cells.** (**A**) Cells treated with different concentrations (0 to 1 mg/ml) of MEGT for 24 h were stained with annexin V-FITC. (**B**) Quantification analysis of annexin V intensity. Data are presented as mean ± S.E. (n = 3). Differences between treatments of different concentrations containing the same letter are not significant.

To further examine whether MEGT-induced sub-G1 accumulation of Ca9-22 oral cancer cells involves apoptosis, the flow cytometry based-annexin V measurement was performed. In Figure 3A, the percentages and profiles of annexin V-positive staining were displayed for the treatments with vehicle control or 0.1, 0.25, 0.5 and 1 mg/ml of MEGT for 24 h. After 24 h MEGT treatment, the annexin V-positive staining of Ca9-22 oral cancer cells was significantly increased in a dose–response manner (*p* < 0.001) (Figure 3).

### Induction of DNA double strand breaks in MEGT-treated Ca9-22 oral cancer cells

**Figure 4:**
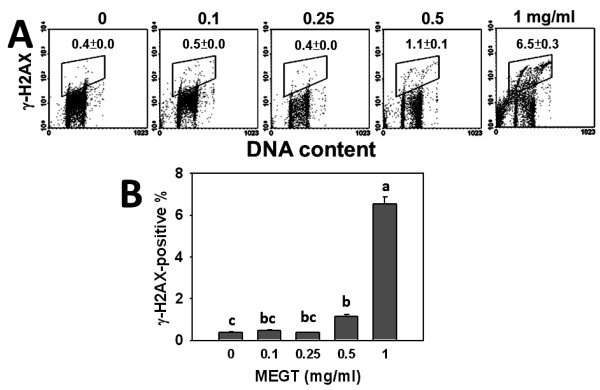
**Methanolic extracts of *G. tenuistipitata *(MEGT) induced DNA double strand breaks of Ca9-22 oral cancer cells.** (**A**) Cells treated with different concentrations (0 to 1 mg/ml) of MEGT for 24 h were stained with γH2AX. (**B**) Quantification analysis of γH2AX intensity. Data are presented as mean ± S.E. (n = 3). Differences between treatments of different concentrations containing the same letter are not significant.

To evaluate whether DNA damage was involved in the MEGT-induced growth inhibition of Ca9-22 oral cancer cells, the flow cytometry based-γH2AX measurement was performed. The percentages and profiles of γH2AX-positive staining were displayed for the treatments with vehicle control or 0.1, 0.25, 0.5 and 1 mg/ml of MEGT for 24 h (Figure 4A). After 24 h MEGT treatment, the γH2AX-positive staining of Ca9-22 oral cancer cells was significantly increased in a dose–response manner (*p* < 0.05) (Figure 4B).

### ROS induction in MEGT-treated Ca9-22 oral cancer cells

**Figure 5:**
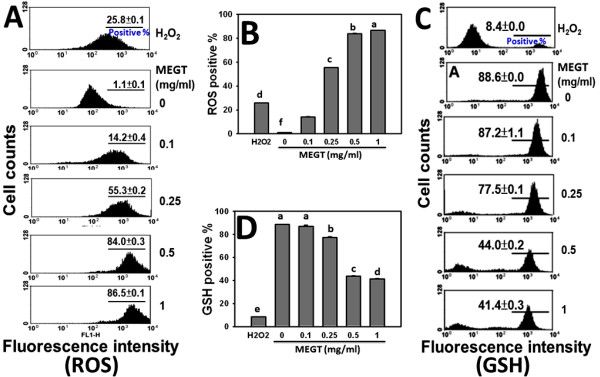
**Methanolic extracts of *G. tenuistipitata *(MEGT) induced reactive oxygen species (ROS) changes and glutathione (GSH) depletion of Ca9-22 oral cancer cells.** (**A**) Flow cytometry-based ROS profiles for MEGT-treated cells. Cells treated with different concentrations (0 to 1 mg/ml) of MEGT and H_2_O_2_ (100 μM; positive control) for 24 h. (**B**) Quantification analysis of ROS intensity. Data are presented as mean ± S.E. (n = 3). (**C**) Flow cytometry-based GSH profiles for MEGT-treated cells. Cells treated with different concentrations (0 to 1 mg/ml) of MEGT and H_2_O_2_ (100 μM; positive control) for 24 h. (**D**) Quantification analysis of intracellular GSH intensity. Data are presented as mean ± S.E. (n = 3). Differences between treatments of different concentrations containing the same letter are not significant.

To evaluate whether ROS was involved in the MEGT-induced apoptosis of Ca9-22 oral cancer cells, the flow cytometry based-DCFH-DA assay was performed (Figure 5). The fluorescence intensities of ROS-positive staining were calculated for various concentrations of MEGT treatment with 100 μM H_2_O_2_ as the positive control. The percentages and profiles of ROS-positive staining of 0, 0.1, 0.25, 5 and 1 mg/ml MEGT for 24 h were calculated (Figure 5A). The ROS-positive intensity of MEGT-treated Ca9-22 oral cancer cells was significantly increased in a dose–response manner (*p* < 0.0001) (Figure 5B).

### Depletion of intracellular GSH induced by MEGT-treated Ca9-22 oral cancer cells

To evaluate whether GSH was involved in the MEGT-induced ROS change of Ca9-22 oral cancer cells, the flow cytometry based-CMF-DA assay was performed. The percentages and profiles of GSH-positive staining of 0, 0.1, 0.25, 0.5 and 1 mg/ml MEGT for 24 h were calculated (Figure 5C). This revealed GSH-positive intensity of MEGT-treated Ca9-22 oral cancer cells was significantly decreased in a dose–response manner (*p* < 0.05) (Figure 5D).

### Reduction of mitochondrial membrane potential (MMP) in MEGT-treated Ca9-22 oral cancer cells

**Figure 6:**
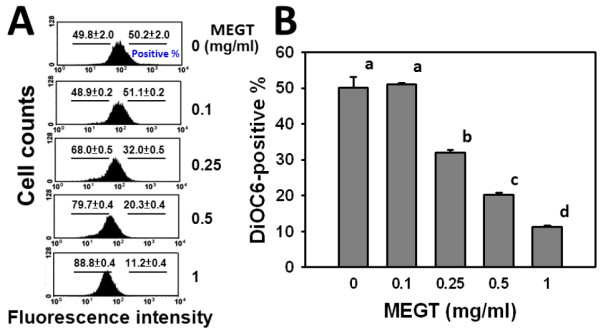
**Methanolic extracts of *G. tenuistipitata *(MEGT) induced mitochondrial membrane potential (MMP) changes of Ca9-22 oral cancer cells.** (**A**) Flow cytometry-based MMP profiles for MEGT-treated cells. Cells treated with different concentrations (0 to 1 mg/ml) of MEGT for 24 h. (**B**) Quantification analysis of DiOC_2_(3)-intensity for MMP. Data are presented as mean ± S.E. (n = 3). Differences between treatments of different concentrations containing the same letter are not significant.

To evaluate whether MMP was involved in the MEGT-induced ROS change of Ca9-22 oral cancer cells, the flow cytometry based-DiOC_2_(3) assay was performed. The percentages and profiles of DiOC_2_(3)-positive staining of 0, 0.1, 0.25, 0.5 and 1 mg/ml MEGT for 24 h were calculated (Figure 6A). This demonstrated the DiOC_2_(3)-positive intensity of MEGT-treated Ca9-22 oral cancer cells was significantly decreased in a dose–response manner (*p* < 0.05) (Figure 6B).

## Discussion

Many genotoxic drugs express their anticancer effects through direct or indirect DNA damage and thus resulting in cell death [37]. Therefore, many chemopreventive agents focus on targeting apoptosis-inducing pathways [38]. For example, several chemotherapeutic drugs, such as cisplatin [39], 5-fluorouracil [40], and taxol [41], have been used for head and neck cancers and are found to induce specific apoptotic pathways. Moreover, drug resistance is partly explained by the ability of cancer cells to evade apoptosis [42-44]. Accordingly, drugs with apoptosis-inducing ability in OSCC may reduce potential drug resistance.

This study shows a novel finding that MEGT has a cytotoxic effect on Ca9-22 oral cancer cells. The cell proliferation inhibiting effect of MEGT in Ca9-22 oral cancer cells is dose-dependent. The IC_50_ value of MEGT-treated Ca9-22 oral cancer cell line is 326 μg/ml (24 h). For comparison, the reported IC_50_ values (treatment times) of water/ethanol/methanol/ethyl acetate extracts for several natural product studies based on cancer cells were found in similar ranges: *Moringa oleifera* leaf water extract for HeLa derivative KB cells [45] was 150 μg/ml (48 h) [46]; *Cassia tora* methanolic leaf extract for HeLa cells was 191 μg/ml (48 h) [28]; *Olea europaea* ethanolic extract for leukemic (Jurkat) cells was 0.9 mg dw (48 h) [29]; *Indigofera linnaei* Ali methanolic extract for cervical, liver, breast, and colon cancer (HeLa, HepG2, MCF-7, and HT-29) cells were less than 100 μg/ml (72 h) [31]; and *Colpomenia sinuosa*, *Halimeda discoidae*, and *Galaxaura oblongata* ethyl acetate extract for liver cancer (HuH-7) cells were 112.38, 230.53, and 123.54 μg/ml (72 h) and for leukemia (HL-60) cells were 53.35, 226.35, and 132.73 μg/ml, respectively.

In experiments with cancer cells other than oral cancer, the incubation times differ from the 24 h incubation period used in this study. Accordingly, the results of lower IC_50_ doses obtained in these studies may be due to the longer incubation times used. In general, the treated dosage of the current developed MEGT is similar to the range of the dosage of natural product-derived extracts used in these cancer studies.

Moreover, the IC_50_ value of clinical chemotherapeutic drug cisplatin was 174 μg/ml (48 h) for HeLa cells [28]. Accordingly, these results suggest that the MEGT is a potential natural product to be used as a chemotherapeutic agent as determined by its effect on Ca9-22 oral cancer cells. Moreover, more natural products with anti-oral cancer activity may be examined for their possible synergistic effects to enhance the efficiency of OSCC chemotherapy.

The possible mechanism for MEGT-induced cell growth inhibition may be partly due to apoptosis in OSCC cells demonstrated by the increases seen using sub-G1 and annexin V-staining. To further validate the role of apoptosis, the caspase signaling pathway [47,48] may need to be examined. The increase of γ-H2AX-based DNA double strand breaks [49] under MEGT administration suggested that DNA damage is involved in MEGT-induced apoptosis. To monitor the γ-H2AX foci [50] and the tailing degrees of the images in single cell electrophoresis (comet assay) [51] by fluorescent microscopy, the evidence of DNA damage may be further observed. Furthermore, the DNA damage response cascade and ROS signaling pathway are two of the main pathways leading to cell death [52,53]. DNA damage has also been reported to induce ROS generation through the H2AX-Nox1/Rac1 pathway [52]. ROS is an important mediator of apoptosis [54] and cell cycle checkpoint functions [55]. Accordingly, the MEGT-induced intracellular ROS changes were examined in OSCC cells.

Many anti-cancer drugs target cells, at least in part, by generating high levels of intracellular ROS [56,57]. In the example of brown algae, ethyl acetate extracts from *Colpomenia sinuosa* were reported to induce apoptosis and intracellular ROS in leukemia U937 cells [31]. Consistently, the current study demonstrated that MEGT induced the ROS in oral cancer cells in a dose–response manner.

In the study of ethyl acetate extracts from brown algae *Colpomenia sinuosa*[31], the research focused on the sub-G1 phase as an indication of apoptosis without further exploring the overall alterations in the cell cycle. In contrast, ethanol extracts of blue-green algae *Aphanizomenon flos-aquae* displayed the G0/G1 arrest in HL-60 leukemia cancer cell lines in 24 h incubation [58]. Similarly, we found that the MEGT-treated Ca9-22 cells at the concentration of 0.25 mg/ml significantly increased the percentage of G1 phase cells and concurrently decreased the percentages of S phase cells and cells following G2/M, supporting the hypothesis that MEGT may lead to a G1 phase arrest before apoptosis. Therefore, algal extract may elicit cell cycle arrest responses.

GSH is protective against intracellular ROS, which induces apoptosis in response to cell injury. Depletion of GSH increases susceptibility to ROS, and results in damage [59] such as DNA fragmentation [60]. Accordingly, many chemotherapeutic agents are developed by the apoptosis-inducible strategy through ROS-mediated cell damage. Consistently, the ROS level of Ca9-22 oral cancer cells after MEGT treatment for 24 h was increased, suggesting that MEGT may induce apoptosis through increasing intracellular ROS and GSH depletion.

In addition, mitochondria also play an important role in ROS and apoptotic events [61]. For example, capsaicin, the main capsaicinoid found in chili peppers, was found to disrupt mitochondrial membrane potential (MMP) and mediate oxidative stress leading to apoptosis in pancreatic cancer cells [62]. Mitomycin c can kill the small cell lung cancer cells by increasing MMP and decreasing intracellular GSH contents due to oxidative stress [63]. The current study demonstrated that MEGT significantly decreased the MMP in oral cancer cells in a dose–response manner, suggesting that MEGT-induced MMP disruption ability may be directly or indirectly influencing ROS generation in oral cancer cells.

Extracts derived from natural products isolated by different solvents may have different cytotoxic efficacies. For example, the IC_50_ value of ethanol extract of *G. tenuistipitata* in Ca9-22 cells was 8-fold higher than that of the methanol extract (data not shown), whereas the water extract of *G. tenuistipitata* did not display any cytotoxicity to H1299 cells [25]. Therefore, methanol extracts of *G. tenuistipitata* were chosen in this study. Although our results have demonstrated the anti-proliferative effect of MEGT on oral cancer cells, there are some limitations in this study. For example, it has been reported that the polysaccharides from the *G. dura*[64], the chemical composition of *G. cervicornis*[65], and the prostaglandin content of *G. verrucosa*[66] are subject to seasonal variation. Accordingly, the possibility of a seasonal variation in the biological effects of MEGT cannot be excluded.

Phenolics are the most abundant secondary metabolites of plants. Methanol is generally efficient for the extraction of lower molecular weight polyphenols [67]. Preliminary analysis indicated that MEGT were rich in polysaccharides and polyphenols (data not shown). The anticancer activities of polyphenols, such as pro-apoptotic and DNA damaging effects have been reported in many literatures [68-71]. Therefore, we expect that the polyphenols are the candidates for the active principles in MEGT which warrants further investigation.

## Conclusion

In this study, the effects of MEGT on an oral cancer cell model were assayed to explore the genotoxic effect, by cell cycle distribution and annexin V detection to explore its apoptotic effect, by γ-H2AX detection to explore its DNA damage effect, by determination of ROS and GSH levels to explore its oxidative stress modifying effect, and by DiOC_2_(3) staining to explore the MMP depolarization. The results of this study have demonstrated that MEGT induced potent anti‒oral cancer effects thought the induction of apoptosis, DNA damage, and oxidative stress pathways. Based on this finding, MEGT may be a potential natural product for anti-oral cancer treatment.

## Competing interests

The authors declare that they have no competing interests.

## Authors’ contributions

C-CY and Y-CC carried out all cell-based experiments. J-IY provided the sample collection and advised C-CY for technical support on extraction. J-CL, C-NT and Y-CH have made substantial contributions to conception and designed the study. C-CY, C-NT, and H-WC drafted the manuscript. J-YT and L-YC performed data analysis. H-WH performed statistical analysis. F-RC and H-WC coordinated, oversaw the study and revised it critically for important intellectual content and corresponding authors. All authors read and approved the final manuscript.

## Pre-publication history

The pre-publication history for this paper can be accessed here:

http://www.biomedcentral.com/1472-6882/12/142/prepub
